# A Comparison between Different Agro-wastes and Carbon Nanotubes for Removal of Sarafloxacin from Wastewater: Kinetics and Equilibrium Studies

**DOI:** 10.3390/molecules25225429

**Published:** 2020-11-19

**Authors:** Marwa El-Azazy, Ahmed S. El-Shafie, Ahmed Elgendy, Ahmed A. Issa, Saeed Al-Meer, Khalid A. Al-Saad

**Affiliations:** Department of Chemistry and Earth Sciences, College of Arts and Sciences, Qatar University, Doha 2713, Qatar; aelshafie@qu.edu.qa (A.S.E.-S.); ae1304231@student.qu.edu.qa (A.E.); ahmedissa@qu.edu.qa (A.A.I.); salmeer@qu.edu.qa (S.A.-M.); kalsaad@qu.edu.qa (K.A.A.-S.)

**Keywords:** waste-derived materials, pistachio nutshells, *Aloe vera* leaves, multi-walled carbon nanotubes (MWCNTs), sarafloxacin

## Abstract

In the current study, eco-structured and efficient removal of the veterinary fluoroquinolone antibiotic sarafloxacin (SARA) from wastewater has been explored. The adsorptive power of four agro-wastes (AWs) derived from pistachio nutshells (PNS) and *Aloe vera* leaves (AV) as well as the multi-walled carbon nanotubes (MWCNTs) has been assessed. Adsorbent derived from raw pistachio nutshells (RPNS) was the most efficient among the four tested AWs (%removal ‘%R’ = 82.39%), while MWCNTs showed the best adsorptive power amongst the five adsorbents (%R = 96.20%). Plackett-Burman design (PBD) was used to optimize the adsorption process. Two responses (‘%R’ and adsorption capacity ‘*q_e_*’) were optimized as a function of four variables (pH, adsorbent dose ‘AD’ (dose of RPNS and MWCNTs), adsorbate concentration [SARA] and contact time ‘CT’). The effect of pH was similar for both RPNS and MWCNTs. Morphological and textural characterization of the tested adsorbents was carried out using FT-IR spectroscopy, SEM and BET analyses. Conversion of waste-derived materials into carbonaceous material was investigated by Raman spectroscopy. Equilibrium studies showed that Freundlich isotherm is the most suitable isotherm to describe the adsorption of SARA onto RPNS. Kinetics’ investigation shows that the adsorption of SARA onto RPNS follows a pseudo-second order (PSO) model.

## 1. Introduction

Environmental pollution has reached worrying levels. Water pollution, specifically, is one of the most critical threats that living beings have ever faced, if not the most challenging of all. The extensive use of antibiotics within either a therapeutic or veterinary context has resulted in a crisis in the long run. Antibiotics can reach the water systems via three major routes: (1) drug production sites, (2) run-offs from the point-of-care locations and the wastewater treatment plants (WWTPs); and (3) the improper disposal of pharmaceutically active materials, including human and veterinary medication residues, as well as the personal care products [1,2]. If existing even as traces, the occurrence of antibiotics in wastewater is responsible for several ecological and health problems [3,4,5].

**Figure 1:**
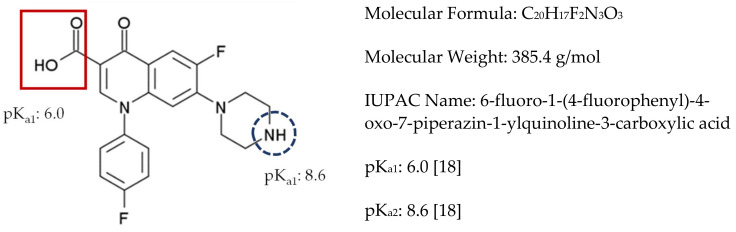
Chemical structure of SARA together with the physicochemical relevant data.

Fluoroquinolone antibiotics (FQs) represent an enormous category of antibiotics with a common bicyclic core (quinolone structure) and a fluorine atom. FQs are active against both Gram positive and Gram-negative bacteria [6]. Being the most detectable category of antimicrobials in water, FQs’ occurrence, fate and removal have been widely investigated [7,8,9,10,11,12]. Sarafloxacin (SARA, Figure 1) is a FQ antibiotic that acts by preventing the activity of DNA gyrase and is used for the treatment of bacterial infections caused by *Escherichia coli* in poultry, pigs, rabbits and dogs. SARA is also used for the treatment of cases of furunculosis, vibriosis and the enteric redmouth disease in *Salmonidae* [13,14,15].

Traces of SARA in the aquatic environment were attributed to animal excretions. SARA was reported to be a persistent antibacterial agent that exists mainly in the deeper layers of the sediment even after 180 days, an issue that implies a resistance to degradation [16,17]. The negative impact of existence of SARA in the aquatic systems is not only restricted to the health of humans and animals, but on the long run, it might result in the development of new strains of antibiotic-resistant microorganisms.

Therefore, finding a suitable approach for removal of SARA is crucial. Many investigations for removal of SARA from contaminated samples were reported using different approaches including mainly photocatalytic degradation, photolysis, adsorption and combinations of these approaches [17,18,19,20,21,22,23,24,25]. Being simple and easy to manipulate with the opportunity of being performed utilizing available materials with no production of toxic byproducts, adsorption is usually seen as one of the most promising approaches for wastewater treatment. Common adsorbents include conventional materials (e.g., commercially available activated carbons and alumina), natural materials (e.g., clay and sand), agro-wastes (AWs) both in their raw format and as activated carbons (ACs) following thermal treatment, hybrid materials, industrial waste products, graphene, carbon xerogels, and carbon nanotubes (CNTs) [2,20,21,22,23,24,25,26,27,28,29,30,31,32,33,34,35].

AWs are composed mainly of lignin and cellulose. These two components are rich in a variety of functional groups (e.g., hydroxyl moieties) and an elevated carbon content. Moreover, AWs possess favorable properties such as high surface area, pore size and volume, and hence are considered as ideal adsorbents for variety of contaminants. On the other hand, adsorbents such as CNTs possess remarkable physicochemical properties, such as a compact structure with a possibility for surface modification, an issue that enables their use for depollution of wastewater [2,36,37,38]. Table 1 shows a summary of the adsorption-based approaches for the removal of SARA from aqueous solutions, spiked seawater and wastewater together with the achieved %removal (%R), adsorption capacity (*q_e_*, mg/g) and the reported kinetics and isotherm models.

Nonetheless, the use of adsorption as a removal approach and the development of novel adsorbents for treating FQs’ contaminated water samples (especially when SARA is the contaminant) might not be as anticipated. Moreover, optimized removal of FQs using multivariate-based approaches is still in a need for major improvements [24,39,40]. Therefore, in the current approach, adsorptive removal of SARA from artificially contaminated water samples and utilizing the adsorptive power of two AWs (pistachio nutshells ‘PNS’ and *Aloe vera* leaves ‘AV’) both in their raw format and following thermal treatment at 500 °C as well as multi-walled carbon nanotubes (MWCNTs) will be the target. Offering compelling advantages in terms of saving time, resources, and minimizing waste with a superb quality of data, coupling of the environmental bioremediation to a multivariate-based statistical platform should be replacing the traditional univariate-based approaches. 

As can be observed form Table 1, most of the reported approaches, if not all, were based on using mainly synthetic or commercially available materials. Moreover, most of these approaches were univariate based. Therefore, the novelty of this approach and to the best of our knowledge stems from using a factorial design for maximizing the adsorptive power of upcycled naturally occurring AWs as well as MWCNTs. Utilization of AWs as waste removers hits two targets; bioremediation of wastewater and protecting the environment from tons of wastes that would represent a burden if not properly disposed.

Plackett-Burman design (PBD) will be the factorial design of choice to evaluate and hence optimize the four variables affecting the adsorption of SARA from the tested samples. Achieving the highest %R and maximizing *q_e_*(mg/g) of the selected adsorbent(s) will be the chief targets [41,42,43]. An assessment of surface properties of the five adsorbents in terms of texture, surface area, pore size and volume, and existence of functional moieties will be performed using the respective characterization approach (Fourier transform infrared (FT-IR) and Raman spectroscopies, scanning electron microscopy (SEM) and Brunauer-Emmett-Teller (BET) analyses). Thermal properties of tested adsorbents will be explored using thermogravimetric analysis (TGA). Isotherms and kinetics of adsorption of SARA will be investigated using the appropriate models.

## 2. Results

### 2.1. Assessment of the Adsorptive Power of the Tested Adsorbents

An initial assessment of the performance of the four adsorbents (obtained from AWs) was gauged using two measures: %R and *q_e_* and employing Equations (1) and (2), respectively. Table 2 reveals the comparison conclusions under the same conditions. As per the displayed results, RPNS had the best adsorptive power among the tested AWs as indicated by the values of %R and *q_e_*. Consequently, RPNS will be further utilized in the supervening investigations and its performance will be further optimized using PBD. Similarly, the performance of MWCNTs as adsorbent will be optimized using the same design:

(1) $$\left(\%R\right)=\;\frac{C_{0}-C_{e}}{C_{0}}\;\times \;100\%,$$

(2) $$\;\;\;\;\left(q_{e}\right)=\;\frac{C_{0}-C_{e}}{W}\;V,$$

where $C_{0}$ (mg L^−1^) denotes the initial concentration of [SARA] solution, $C_{e}$ is the concentration of the [SARA] solution at equilibrium, V stands to the volume of the solution (L), and W is the weight of the adsorbent used (g).

### 2.2. Factorial Design

PBD was chosen to optimize the measured responses (%R and *q_e_*) as a function of the four variables. In addition to the advantages of being a multivariate approach, PBD offers other pros such as detecting the statistically significant variables from a relatively large number of factors affecting a process (2–47 variables). Moreover, and in addition to being an efficient approach when only main variables are concerned, PBD is one of the most commonly used approaches for testing method robustness [28,29,41,42,43]. The proposed experimental setup is shown in Table 3 in terms of coded/un-coded variables together with the variable levels (higher, lower and mid-levels). The detailed experimental scenario is shown in Table 4 together with measured values of both responses and the predicted values as calculated by Minitab^®^19.

### 2.3. Assessment of Statistically Significant Independent Variables

**Figure 2:**
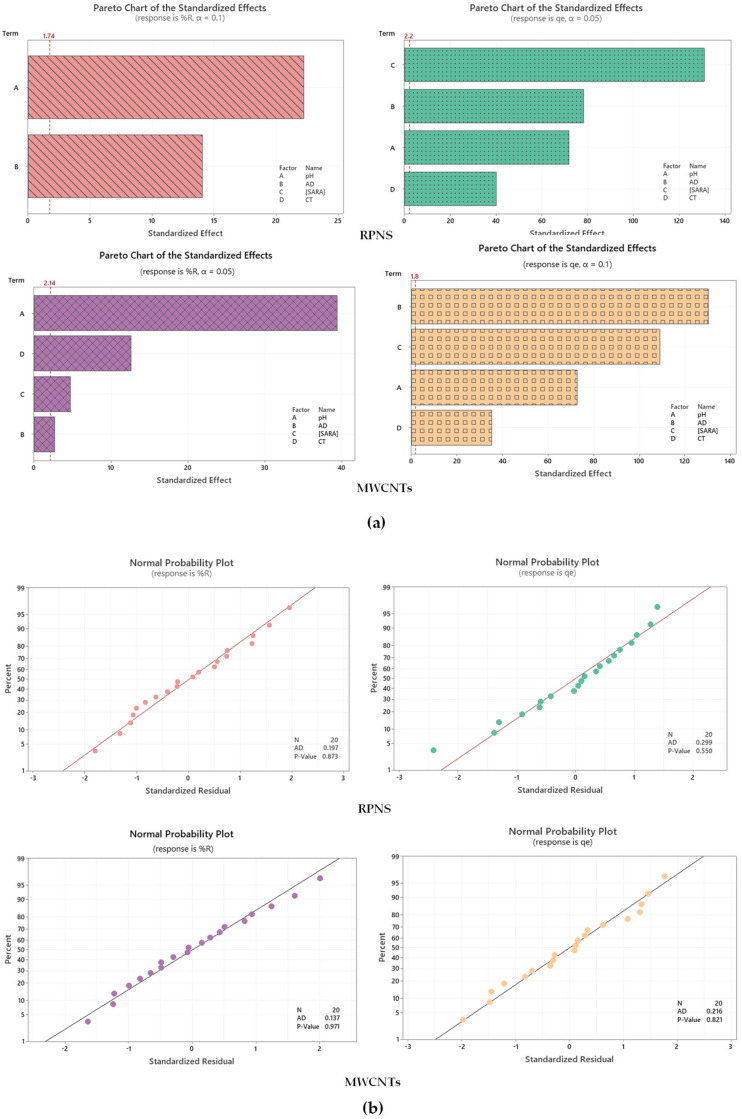
(**a**) Pareto chart of standardized effects; and (**b**) Probability plots for the two measured responses. For each panel, %R is shown on the Left while *q_e_* is the Right panel. Represented data were obtained following response transformation.

Statistical significance of the tested variables (shown in Table 3 and Table 4) was assessed using a combination of quality charts (mainly Pareto chart of standardized effects to denote the magnitude of each variable, Figure 2) in conjunction with the analysis of variance (ANOVA), Table 5. As revealed in Figure 2a—upper left panel where RPNS is the tested adsorbent and the %R is the measured response, pH(A) was the most statistically significant variable, followed by the dose of RPNS(B). Other charts such as normal plot of standardized effects was used to decide upon both the magnitude and the direction of the studied factors—Figures are not shown. Results from these graphs show that the pH has a negative influence on the %R in case of RPNS. In case of adsorption capacity (*q_e_*) being the measured response—Figure 2a—upper right panel, all the four variables were statistically significant with [SARA] being the most influencing variable followed by the effect of the dose of RPNS. The situation is a bit different using MWCNTs as an adsorbent, where %R is mainly affected by pH(A) and CT (D) and *q_e_* is mainly influenced by AD (B) and [SARA].

Normal probability plots as an example for residual plots are shown in the lower panel (Figure 2b) for both adsorbents. As shown, the *p*-value was >0.05 and the Anderson-Darling statistic (A–D) was relatively low, implying that obtained data follow a normal distribution [44].

Findings of the ANOVA testing at 95.0 confidence interval (95.0 CI) further confirm the previous conclusions for the statistical significance of the assessed variables. As shown in Table 5, the magnitude of the F-value increases with the increase in the magnitude of the variable’s impact. The significance level (*p*-value) is <0.05 for statistically significant variables. In this itinerary, the lack-of-fit was statistically insignificant with a *p*-value >0.05 insinuating the good fit. It is essential to state that regression of measured responses was done versus central points (Ct Pt), together with assessed variables using Box-Cox response transformation [45]. Stepwise analysis implementing backward elimination of terms (α to remove = 0.1) was used in case of %R. The regression outcome is shown in Equations (3)–(6):

√%R_(RPNS)_ = 9.852 − 0.5996 pH + 0.02525 AD, (3)

*q_e_*^0.45868_(RPNS)_ = 1.9552 − 0.11712 pH − 0.008492 AD + 0.028427 [SARA] + 0.002375 CT − 0.11314 Ct Pt, (4)

%R^1.5_(MWCNTs)_= 1450.5 − 134.11 pH + 0.607 AD − 2.154 [SARA] + 1.564 CT + 2.1 Ct Pt, (5)

ln(*q_e_*)_(MWCNTs)_ = 2.4965 − 0.19256 pH − 0.023006 AD + 0.038492 [SARA] + 0.003400 CT + 0.09626 Ct Pt, (6)

Regression equations (Equations (3)–(6)) confirm the previously mentioned effects of the tested variables in terms of magnitude and direction. For example, the impact of pH on both responses, %R and *q_e_* (for each adsorbent) was similar, in contrast to AD which had a positive effect on %R compared to a negative effect in case of *q_e_*.

Summaries of both models exhibited in Equations (3)–(6) show that the coefficient of determination (R^2^) value was comparatively high (R^2^ = 97.60% and 99.96% in case of RPNS and 99.20% and 99.96% in case of MWCNTs) and akin to the value of R^2^–adjusted (R^2^ adj) = 97.32% and 99.94% in case of RPNS and 98.92% and 99.95% in case of MWCNTs), signifying the linearity of the regression models. The capability of models to predict the new observations was signified by the high values of R^2^–predicted (R^2^ pred = 96.41%, 99.92% in case of RPNS and 98.53%, 99.90% in case of MWCNTs). This conclusion could be further corroborated by the low values of relative error (RE) exhibited in Table 4.

### 2.4. Contour and Surface Plots

**Figure 3:**
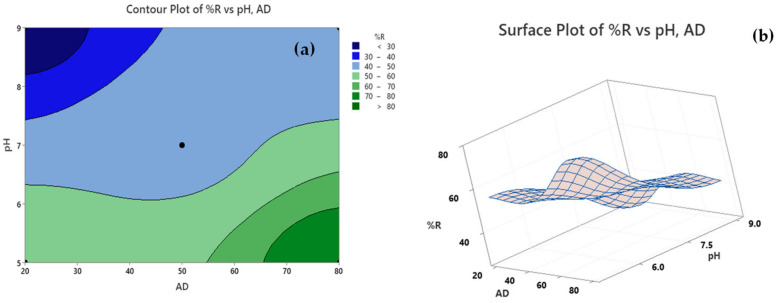
(**a**) Contour and (**b**) Surface plots for %R using RPNS.

An investigation of how the fitted response values correlate to two continuous variables based on the regression equations is symbolized by the contour plot (Figure 3a). Shown plot signifies a 2D—view in which all the points having the same response are associated to produce a contour line and reflect the impact of pH and AD on %R. As per the attached legend, the dark green region denotes a zone where maximum %R can be achieved using an AD of 65–80 mg/50 mL and at pH of 5.0–<6.0. Another approach to establish the relation between the response values and the operating conditions is the 3D—representation of response via surface plots. Compared to the contour plots, surface plots provide a better understanding for the response surface. Figure 3b shows that maximum %R (elevated ridge) can be achieved employing an AD of ~65–80 mg/50 mL and pH value of 5.5–6.0.

### 2.5. Response Optimization

**Figure 4:**
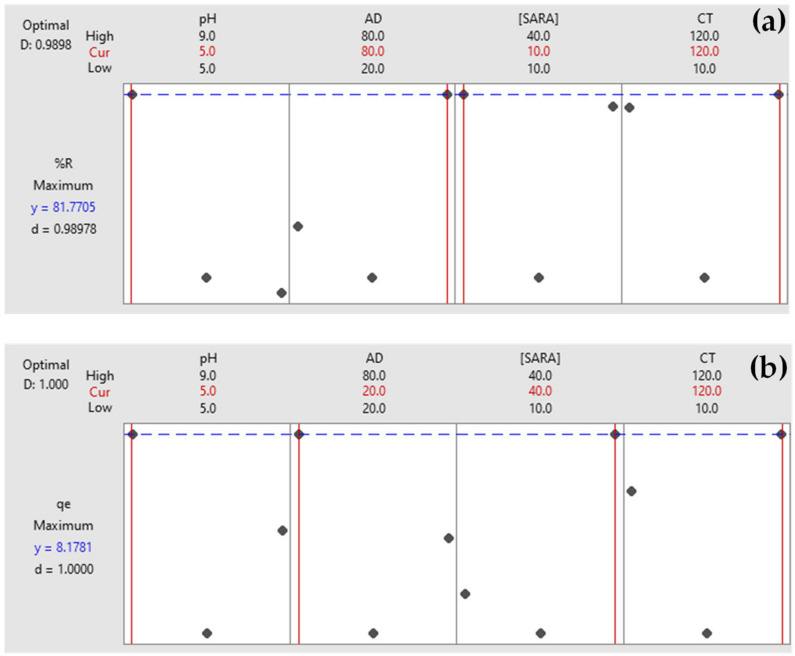
Optimization plots for (**a**) %R and (**b**) *q_e_* using RPNS.

Figure 4 shows optimization plots for both responses using RPNS as an adsorbent. The target was set to maximize each response. Variable limits that achieve the maximum of each response are shown as ‘Cur’. For example, to achieve a %R of 81.77% for a [SARA] = 10 ppm using RPNS, a blend of pH 5.0, AD = 80 mg/50 mL and CT = 120 min. would produce a high desirability value (*d*) = 0.9898, implying the favorability of the selected variable levels [46]. To achieve a %R of 99.64% using MWCNTs, the same blend of variables achieved a desirability of 1.0000. Similarly, to achieve *q_e_* = 8.17 and 20.52 mg/g using RPNS and MWCNTs, respectively, variable limits should be kept at pH 5.0, AD = 20 mg/50 mL and CT = 120 min. for [SARA] = 40 ppm.

### 2.6. Characterization of Tested Adsorbents

#### 2.6.1. Thermal Characteristics

**Figure 5:**
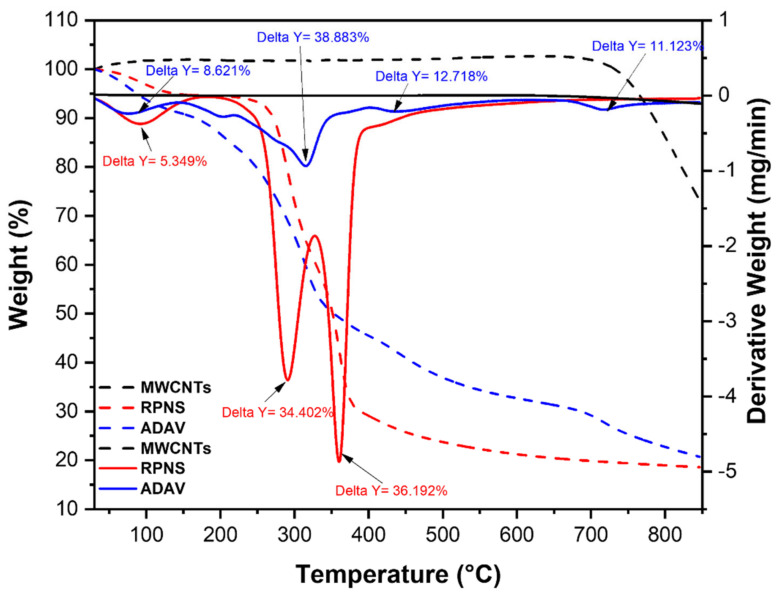
TGA/*d*TA analysis of RPNS, ADAV, and MWCNTs.

Thermal stability of raw AWs and MWCNTs was investigated using thermogravimetric analysis (TGA, Figure 5). TGA analysis shows that MWCNTs are thermally stable and no weight loss could be observed along the temperature range of 100 and up to 700 °C. Weight loss was only observed after 700 °C [47]. This finding implies that the adsorption of SARA onto MWCNTs might occur via physisorption. In case of RPNS, the overall weight loss could be attributed to the loss of mainly oxygen and carbon in RPNS. Multistep decomposition occurs as follows: (1) vaporization of free water (5.349%) at a range of 25–150 °C, (2) loss of crystalline water at ~200 °C, and (3) loss of the organic matter (carbon-based functional groups) at the range of 200–400 °C. In case of ADAV, similar behavior could be observed where water loss was observed at the same range up to 150 °C followed by loss of organic matter up to 600 °C and finally carbonization of the polymeric material up to 800 °C. Findings of TGA, therefore, implies the absence of thermally labile functionalities following carbonization, an issue that explains the diminished adsorptive power of thermally treated AWs compared to the raw biomasses and the possible involvement of chemisorption in case of the former biosorbents [27,28,48,49]. The adsorption mechanism will be further investigated in the next sections.

#### 2.6.2. Functional Groups and Adsorption Mechanism

**Figure 6:**
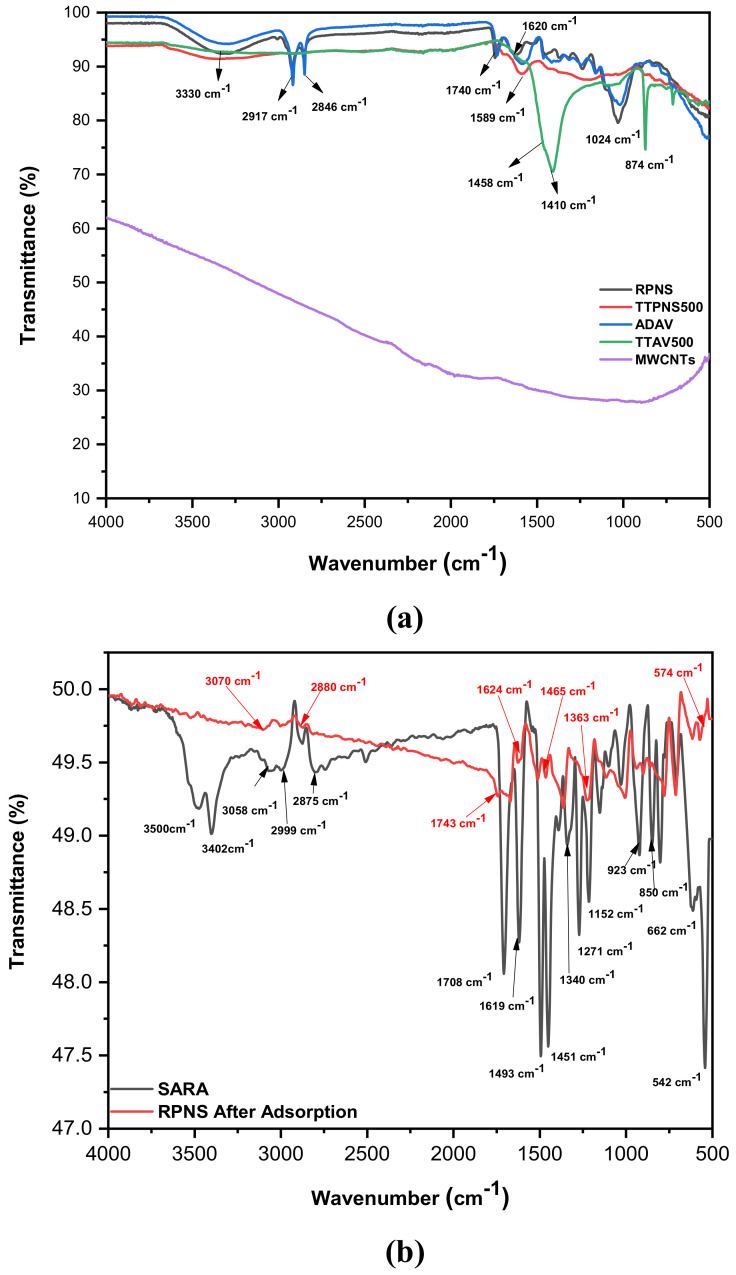

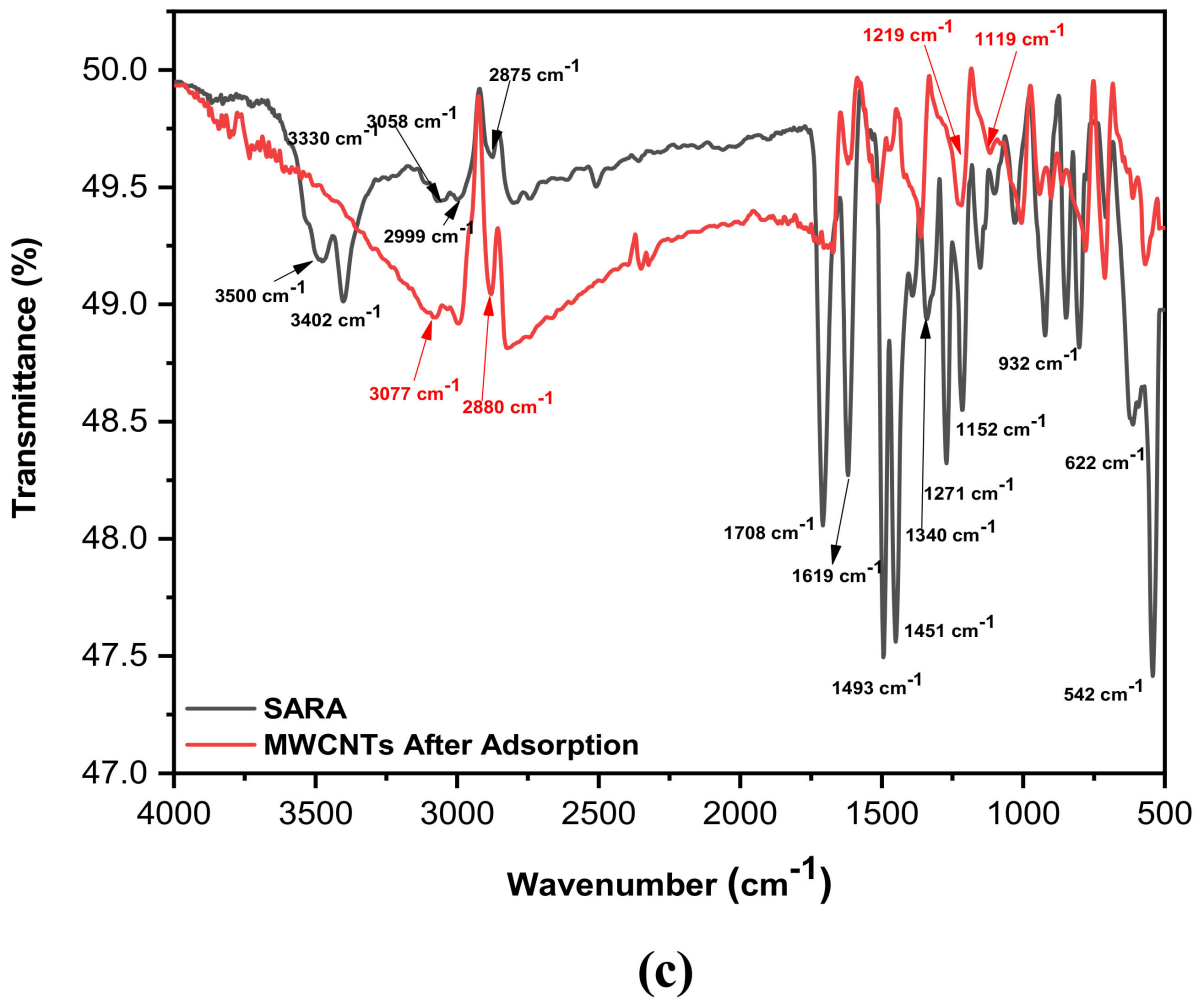
(**a**) FT-IR spectra of tested adsorbents before adsorption, (**b**) SARA and RPNS after adsorption and (**c**) SARA and MWCNTs after adsorption.

The functional groups on the surface of both types of adsorbents (AWs and MWCNTs) were determined using FT-IR. Figure 6a (upper panel) shows a screening of the functional moieties in the five adsorbents before the adsorption of SARA. As previously reported, agricultural biomasses are of a lignocellulosic nature (composed mainly of lignin, cellulose and hemicellulose). With a high content of functional groups (especially hydroxyl either aliphatic or aromatic, carbonyl, carboxyl, and amino functionalities) and an elevated carbon content, lignocellulosic biomasses are considered as ideal adsorbents [50].

Raw (non-thermally treated) biomasses (RPNS and ADAV) show almost identical spectra with different intensities [27,28]. The peak at 3330 cm^−1^ is evidently attributed to the -OH stretching vibrations and signifies the existence of polysaccharides. Existence of chemisorbed water (especially in ADAV) on both cellulose and lignin can also explain the broad peak at 3330 cm^−1^. Both adsorbents showed symmetric and asymmetric C-H stretching vibrations of the lignocellulosic content at 2846 and 2917 cm^−1^ implying the presence of either CH_2_ or CH_3_ groups or both moieties. The peak at 1740 cm^−1^ could be assigned to the carbonyl moiety from esters or lactones. A peak at 1620 cm^−1^ can be assigned to the -OH bending vibration of absorbed water. The peak at 1024 cm^−1^ represents the C-O stretching vibration in lignin, cellulose and hemicellulose [51].

Thermally treated biomasses (TTPNS500 and TTAV500) show a complete disappearance of some peaks and shifting in the positions of others compared to the raw adsorbents. Peak at 1458 cm^−1^ can be attributed to the CH_2_ deformation stretching in lignin and the carbohydrate complex. The sharp peak at 874 cm^−1^ could be assigned to the C–H (out of plane) glucose ring moiety in cellulose and hemicellulose and for the guaiacyl rings in lignin [52,53,54]. MWCNTs, on the other hand, show almost no functionalities. This difference in existence of functional groups was further motivating to study the adsorption mechanism.

On the other hand, SARA ‘the adsorbate’, shows peaks at ~3500 and 3058 cm^−1^ which could be attributed to O-H stretching vibration, intermolecular H-bonded. A prominent peak at 3402 cm^−1^ could be assigned to the N–H stretching vibration of the imino- or the piperazinyl moieties. Peaks at 2999, 2875 cm^−1^ could be assigned to aromatic, cyclic enes (*υ* = CH and Ar-H). The peak at 1708 cm^−1^ can be assigned to the C=O stretching vibration of the carboxylic acid. Peak at 1619 cm^−1^ could be due to N–H bending vibration of the quinolines moiety. The peaks at 1493 and 1451 cm^−1^ might be assigned to the stretching vibration of the O-C-O group. The C-F stretching vibration can be seen at 1000–1152 cm^−1^ [55,56].

Adsorption of SARA onto the surface of RPNS and MWCNTs was investigated after adsorption and was indicated by the appearance of new functionalities on the tested adsorbent’s surface or shifting of the existing functional groups due to chemical bonding or physisorption on the surface, Figure 6b,c. This change was more prominent in case of MWCNTs, where the pristine adsorbent and as indicated had almost no peaks. Figure 6c shows that all peaks of SARA stay the same but with less intensity following the adsorption of SARA onto the surface of MWCNTs.

In case of RPNS, the spectrum of SARA at the fingerprint region 500–1500 cm^−1^ stays the same with less intense peaks following the adsorption onto RPNS. A new weak peak at 3070 cm^−1^ appears after adsorption with almost complete disappearance of the peaks attributed to the O-H stretching vibrations from both SARA and pristine RPNS. Another peak which was attributed to the C=O stretching vibration of the carboxylic acid in SARA and the carbonyl moiety from esters or lactones has shifted to 1743 cm^−1^ implying the involvement of this moiety in the binding process.

**Figure 7:**
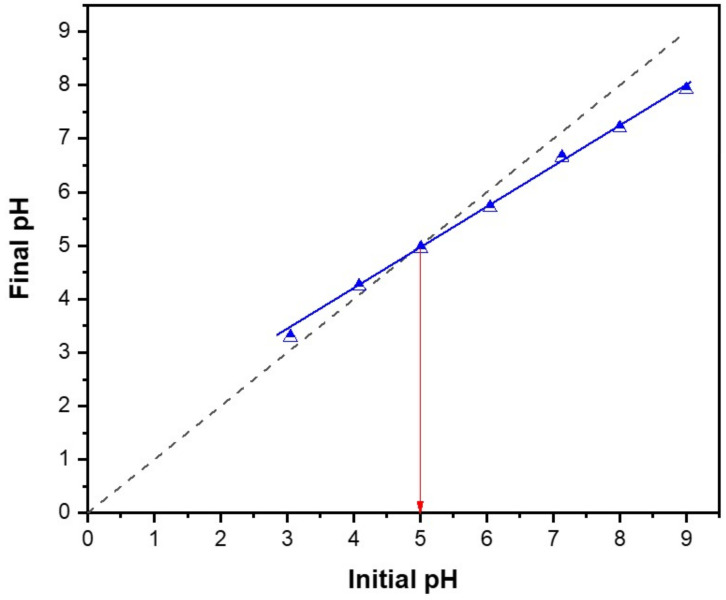
Determination of the pH_pzc_ of the RPNS sample.

As previously reported [18], SARA is of an ampholytic nature with a pK_a_ value of 6.0 for the carboxylic moiety and 8.6 for nitrogen atom in the piperazinyl ring (Figure 1). Therefore, and as seen in Table 3 and Table 4, the impact of pH on the removal power (%R) of RPNS was studied at three levels: 5.0, 7.0 and 9.0 ± 0.2. At a pH value <6.0, SARA exists mainly in the cationic form with a small amount of zwitterionic form. On the other hand, when pH is in the range of 6.0 < pH < 8.6, the three forms of SARA happen together with the zwitterionic form being the major component, and the amount of cationic form subsides with the rise of pH, whereas the amount of anions increases with the increase of pH. When the pH > 8.6, SARA exists mainly in the anionic form, together with a small amount of zwitterionic form. On the other hand, RPNS shows a point-of-zero-charge (PZC) of 5.0 (Figure 7) [57,58,59]. So, at low pH values (<5.0), the adsorbent surface will be positively charged. Therefore, the best adsorption should be attained between pH 5.0–6.0.

#### 2.6.3. Raman Analysis

**Figure 8:**
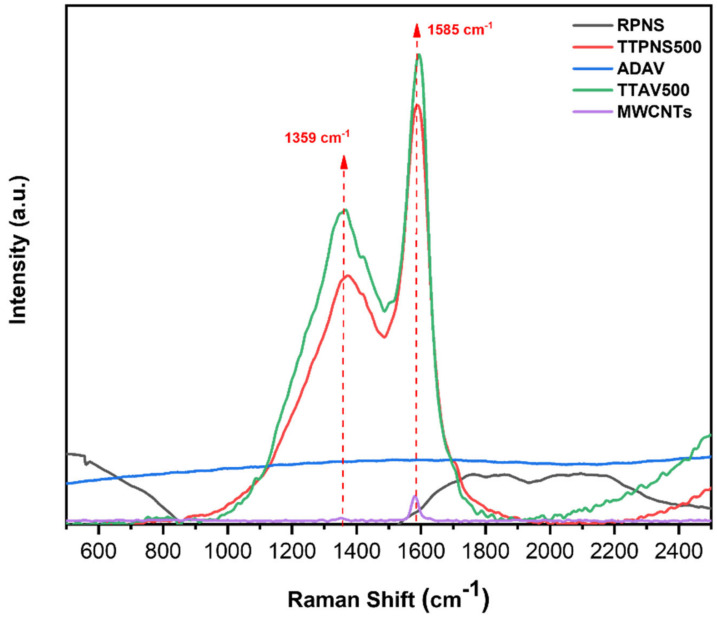
Raman spectra of the tested adsorbents.

Raman spectra of raw, thermally treated biomasses at 500 °C, and MWCNTs are presented in Figure 8. As can be seen from the revealed spectra, only the thermally treated samples and the MWCNTs show two types of bands: a band at 1351 cm^−1^ (D-band) and a band at 1585 cm^−1^ (G-band). On the other hand, the spectra of raw AWs do not show such bands (except for RPNS where the existence of such peaks might be attributed to exposure of nutshells to some sort of thermal treatment before being sold though being labelled non-roasted). The appearance of these two bands in general could be attributed to the carbonaceous nature of the thermally treated AWs and the MWCNTs. While the D-band reflects the characteristics of the carbon lattice including defects and sizes, the G-band detects the stretching of C-C for the sp^2^ system. Nevertheless, the D-band does not signify the chemical structure of the carbon material. Table 6 shows the intensity ratio of I_D_:I_G_ for all five sorbents. As can be seen, the ratio was highest in case of TTAV500 followed by RPNS500 and then the RPNS. This finding implies the conversion of the AWs into carbonaceous material and that the thermal treatment process might have increased the defect states in carbon, a case which is not found in MWCNTs inferring less defects.

#### 2.6.4. Morphology Characteristics

**Figure 9:**
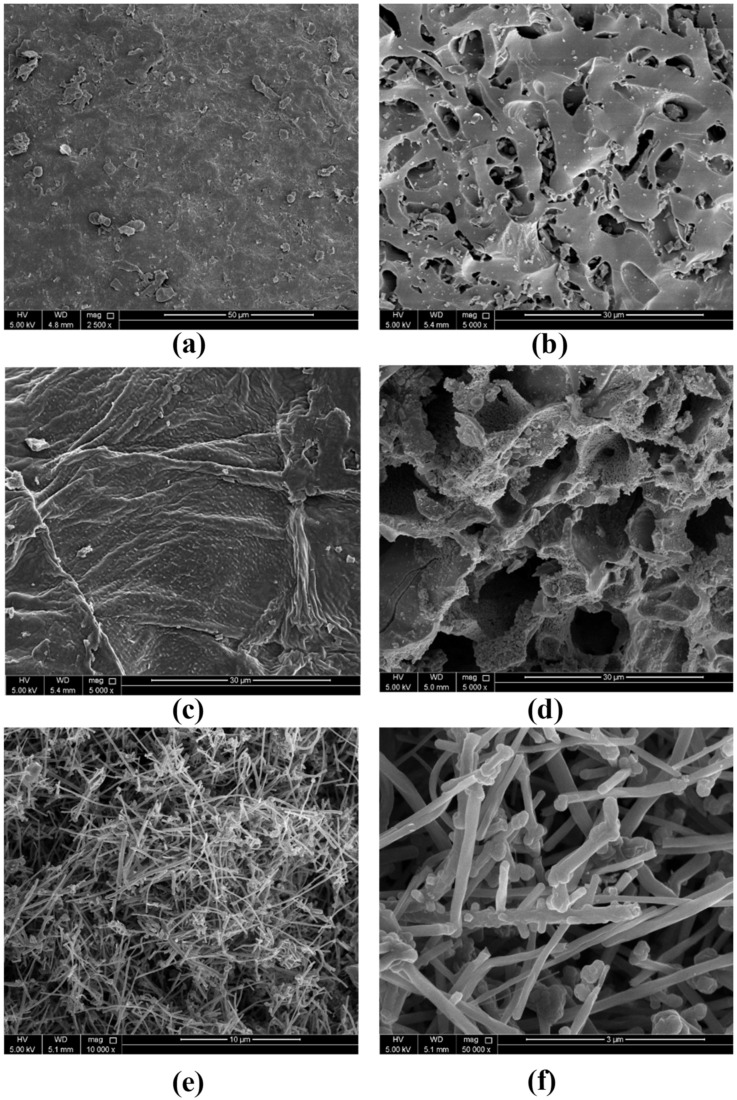
SEM micrographs of (**a**) RPNS, (**b**) TTPNS500, (**c**) ADAV, (**d**) TTAV500, (**e**) MWCNTs 10 µm magnification and (**f**) MWCNTs 3 µm magnification.

**Figure 10:**
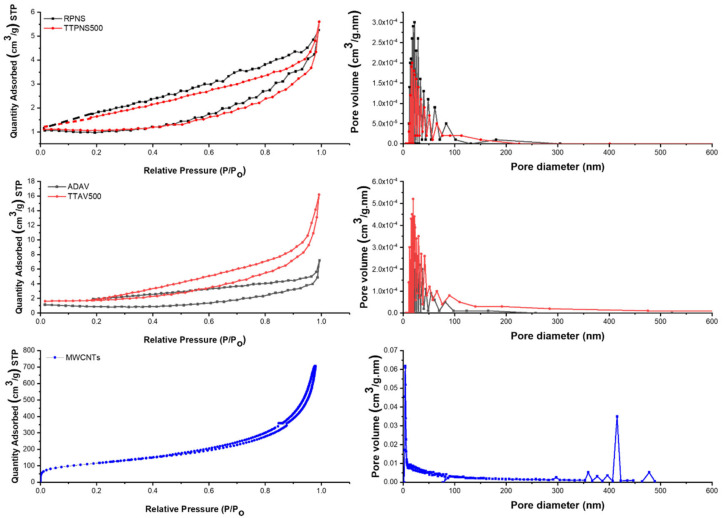
BET analysis of the five adsorbents.

Surface morphological characteristics were investigated using SEM together with BET analyses. Figure 9 shows the SEM micrographs of the five tested adsorbents. A common feature in raw AWs was the smooth meso- to macroporous surface (Figure 10). On the contrary, the thermally treated AWs showed a porous surface with mainly mesopores. Table 6 shows that the surface area (SA) of the MWCNTs was at least six times higher than the highest SA for the AWs and around 122 times higher compared to the SA of ADAV. Similarly, the pore volume for MWCNTs was much higher compared to any of the AWs. Yet, and as shown in Figure 10, MWCNTs has few pores and the exiting pores are mainly macroporous. These findings together with the FT-IR analysis confirm that adsorption of SARA onto RPNS is mainly controlled by the chemical structure of the adsorbent’s surface, in contrast to the MWCNTs where adsorption is controlled by the SA of the adsorbent. 

Figure 10 shows that the BET adsorption isotherm was of type IV in case of MWCNTs, implying the occurrence of monolayer-multilayer adsorption followed by capillary condensation. It is noteworthy to mention that the initial part of type IV is similar to type II which occurs in case of non-porous surfaces like that of MWCNTs. The hysteresis loop is of H3 type inferring the formation of loose masses of plate-like particles forming slit-like pores. Like MWCNTs, the other four adsorbents show type IV BET isotherm. Also, similar to MWCNTs, adsorbents from PNS show H3 loop, while those from AV leaves show H4 loop inferring the existence of narrow slit-like pores as well as particles with internal voids of irregular shape, hollow spheres and broad size distribution [60].

### 2.7. Equilibrium and Kinetics Studies

The data in Table 2 show that RPNS and ADAV have the best removal efficiency for SARA compared to the thermally treated samples. The noteworthy to mention observation is that RPNS and ADAV are raw materials that are rich in C-O-C and C-OH groups as reported in the FT-IR section. Therefore, we expect that there are interactions between SARA and oxygen rich groups, which could be physical due to intermolecular forces or chemical due to the interaction of carboxylic or amine groups in SARA with OH groups on the surface of dried biomass. In this section, we are going to investigate the adsorption isotherms and kinetics.

#### 2.7.1. Equilibrium Isotherms

Adsorption isotherms express the specific relationship between the degree of accumulation on the adsorbent surface and the concentration of the adsorbate at a constant temperature. Langmuir, Freundlich, Temkin and Dubinin-Radushkevich (DR) isotherms have been used in the current investigation to study the adsorption of SARA onto RPNS from an aqueous solution [61,62,63,64]. These four isotherms are used extensively in most of the adsorption investigations.

**Figure 11:**
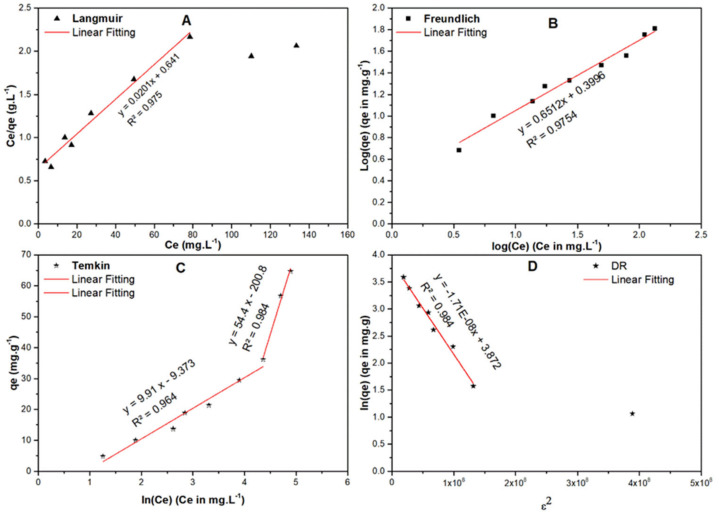
Adsorption isotherms of SARA onto RPNS including (**A**) Langmuir, (**B**) Freundlich, (**C**) Temkin, and (**D**) Dubinin-Radushkevich (DR).

Langmuir isotherm has three assumptions: (I) the adsorption energy is constant across all sites, (II) each molecule occupies only one site and no interaction between the molecules, finally (III) the adsorption is localized. Langmuir isotherm has been represented by Equation (7) and Figure 11a. The later shows a linear response up to a certain limit (80 ppm).

(7) $$q_{e}=\frac{q_{m}\;K_{L}\;C_{e}}{1-K_{L}\;C_{e}}$$

where *q_m_* is the maximum adsorption capacity and *K_L_* is Langmuir equilibrium coefficient. In addition, the Langmuir equation can be stated using the following dimensionless equation:

(8) $$R_{L}=\frac{1}{1+K_{L}\;C_{0}}$$

where *R_L_* is separation factor and C_0_ (mg/L) is the initial concentration. Based on previous reports, the adsorption favorability is dependent on the value of *R_L_*. In general, if *R_L_* is >1, the adsorption process is unfavorable, if *R_L_* = 1, adsorption is linear and with a value between 0–1, the adsorption is favorable (occurs spontaneously) and if it equals zero, adsorption is irreversible. The calculated *R_L_* value in the current investigation was <1 and tends to be zero at high concentration, implying that the adsorption process was spontaneous and at high concentration becomes irreversible with maximum adsorption (*q_max_*) = 49.75 mg/g.

The Freundlich isotherm is a pure empirical approach and is used to describe heterogeneous surface energies as given by Equation (9):

(9) $$q_{e}=\;K_{F}C_{e}^{\frac{1}{n}}$$

Here, C_e_ is the equilibrium concentration of SARA (mg/L); *q_e_* is the amount of SARA adsorbed/unit mass (mg·g^−1^), K_F_ (mole·g^−1^)(L·mole^−1^)^1/n^ and1/n are Freundlich coefficients (Figure 11b and Table 7). The Freundlich plot (Figure 11b) showed a good fit with an R^2^ = 0.975, 1/n = 0.651 and n = 1.536. Therefore, the adsorption potential (A = nRT) = 3.85 kJ and hence any SARA molecule with a potential energy <3.85 kJ will be adsorbed onto the surface of RPNS and reactions tend to be irreversible and favorable. These data confirm that Freundlich isotherm is more suitable to describe the adsorption process of SARA onto RPNS.

The Temkin isotherm (Figure 11c) gives an idea about the adsorbate-adsorbent interactions. As per this isotherm, the heat of adsorption of all the molecules in the layer decreases linearly with the adsorbent-adsorbate interactions. The data revealed in Table 7 show that there are two regions; the first one is less than equilibrium concentration (90 ppm), in which the sorption energy is 250 J/mol, while the other region is where the concentration is higher than 90 ppm and in which the sorption energy is 45.5 J/mol. This finding implies that the adsorption of SARA onto RPNS goes over two stages; first stage could be attributed to chemical adsorption to form one layer (confirmed by Langmuir isotherm) and the second is attributed to the physical interaction to form multilayers and this could interpret the isotherms of Langmuir and Freundlich.

Finally, the DR isotherm (Figure 11d) is built on the potential theory. The figure shows two regions; one at very low concentration, which could be attributed to ion exchange as revealed by the sorption energy (equals 15.981 kJ/mol), and the other region is physisorption where the sorption energy is 5.423 kJ/mol as shown in Table 7. In addition, the maximum capacity in the very low region is 6.23 mg/g, while the maximum capacity in the other region is 48.04 mg/g which is aligned with Langmuir maximum capacity.

#### 2.7.2. Kinetic Studies

**Figure 12:**
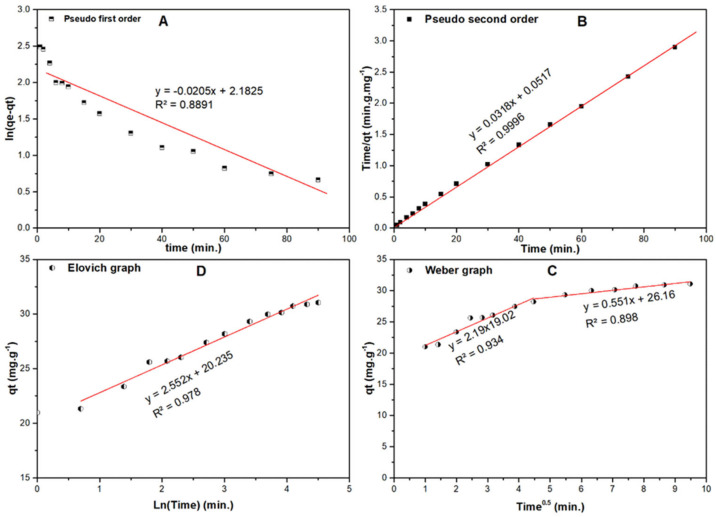
(**A**) First order, (**B**) second order, (**C**) Elovich and (**D**) intra-particle diffusion (WM) curves of adsorption of SARA onto RPNS.

Four kinetic models; namely pseudo-first order (PFO), pseudo-second order (PSO), Elovich and Weber-Morris (WM) were applied to simulate the kinetics of the adsorption of SARA onto RPNS. Figure 12a,b show the plots of ln (*q_e_* − *q_t_*) and time/*q_t_ vs* time for the PFO and PSO kinetic models, respectively. The calculated parameters of the two models are listed in Table 8. By comparing the R^2^ values of the two models, it could be concluded that the experimental data were in good agreement with the PSO model (R^2^ = 0.993). Therefore, the reaction is represented as follows:

(10) $$SARA+RPNS\;\left(\overset{k}{\to }\;\right)\;\;\left\{SARA-RPNS\right\}$$

According to the Elovich model shown in Figure 12c, the initial adsorption is very high 7.1 × 10^3^ mg·g^−1^·min^−1^. The Weber-Morris (WM) intraparticle diffusion model (Figure 12d) reveals many significant facts. First, there is another mechanism that controls the diffusion beside the intra-particle diffusion. Second, the diffusion occurs in two stages, starting with high diffusion rate (=2.19 mg·g^−1^·min^−0.5^) with less boundary layer equals 19.02 mg/g, then the diffusion rate decreases when the boundary layer equals 26.16 mg/g.

## 3. Materials and Methods

### 3.1. Materials and Reagents

All reagents and chemicals were of analytical grade and were used without further purification. Ultrapure water (18.2 MΩ) was used to prepare and dilute needed solutions. SARA and hydrochloric acid were procured from Sigma–Aldrich (Eschenstrasse, Taufkirchen, Germany). Multi-walled carbon nanotubes (MWCNTs, 90% carbon basis, D x L 110 – 170 nm × 5 – 9 µm) was also from Sigma-Aldrich but produced in Japan. Ammonium dihydrogen orthophosphate and sodium hydroxide were purchased from BDH Laboratory Supplies (Poole, UK). Raw adsorbents (RPNS) were purchased from local hypermarkets in Doha-Qatar, while fresh and green AV were collected from a backyard located in Doha, Qatar.

### 3.2. Equilibrium and Kinetic Studies

The equilibrium studies for the adsorption of SARA onto RPNS were done using a 500-ppm stock solution of SARA. Dilutions of the stock solution; 5–350 ppm were prepared in deionized water and the pH was adjusted to pH 5.00 ± 0.20 using phosphate buffer solution. Equal quantities of RPNS (0.050 g ± 0.005) were added to 15 mL of the previously prepared solutions. The prepared solutions were then shaken using an automatic shaker at 150 rpm for 2 h. then filtered. The absorbance of the filtrate was measured at 318 nm. On the other hand, the kinetics studies were done by mixing a 200 mL SARA drug solution (100 ppm, pH 5.00 ± 0.20) and ~1.0 g of RPNS with shaking, and one sample was taken at different time range over a time span of 90 min.

### 3.3. Instrumentation and Software

A diode array UV-Vis spectrophotometer (Agilent, Santa Clara, CA, USA) with 10 mm matched quartz cells was used to measure the absorbance of the aqueous solutions before and after adding the different adsorbents. A ST8 benchtop centrifuge (Thermo Scientific, Waltham, MA, USA) was used to separate the supernatant. The pH of the prepared solutions was adjusted using a Jenway pH meter (Jenway, Staffordshire, UK). Minitab^®^19 software was provided by Minitab Inc. (State College, PA, USA). The software was used to make the list of experiments according to the selected design. FT-IR spectroscopy (Bruker Alpha, Billerica, MA, USA) was used to explore the functional groups on the adsorbent’ surface before and following the adsorption process. Raman spectroscopy (Thermo Scientific, Waltham, MA, USA) was used to examine the formation of carbonaceous materials following the thermal treatment at 500 °C. Surface morphology was examined using scanning electron microscope (SEM, FEI, Quanta 200, Thermo Scientific) and energy dispersive X-ray spectroscopy (EDX, Thermo Scientific). Thermal stability of prepared adsorbents was inspected using a thermal gravimetric analyzer (TGA400, PerkinElmer, Waltham, MA, USA). Thermogravimetric analysis was performed under N_2_ with a heating rate of 10 °C/min. Measurements of surface area, pore size, and volume were performed using a Micromeritics ASAP2020 accelerated surface area and porosimetry system. Degassing of samples was initially applied, followed by studying N_2_ adsorption-desorption. 

### 3.4. Preparation of the AWs’ Derived Adsorbents

As mentioned, pistachios were purchased from the local hypermarkets in Doha-Qatar. The packet label showed that pistachios are non-salted and non-roasted. RPNS and TTPNS500 were prepared as previously reported [27]. Briefly, the green kernels were cleared from the nutshells and the later were rinsed with distilled water several times. Washed nutshells were dried in the oven at 60 °C for five consecutive days, grinded, sieved, and then split into two allotments. The first part was preserved in tightly closed bottles in the desiccator and marked as RPNS. The other part was burnt in the oven at 500 °C and labelled as TTPNS500. Similarly, ADAV and TTAV500 were prepared as previously described [28]. In brief, the collected leaves were washed several times with water. The insider gel was taken off and leaves were re-washed again with distilled water. Leaves were cut into smaller pieces and left in shade for 5 days. Further oven drying was made at 50 °C for 2 h. Dry leaves were then pulverized and divided into two parts; the first is kept in sealed containers and labelled as ADAV. The second was exposed to thermal treatment at 500 °C for 2 h and labelled as TTAV500.

### 3.5. Determination of the Point of Zero Charge of RPNS

The pH drift method was used in this work to determine the point of zero charge (pH_PZC_) of the RPNS sample [56,57,58]. The samples were prepared by adding 1.0 g of RPNS to each flask of seven Erlenmeyer flasks containing 50 mL of 0.01 M NaCl. The pH within each flask was adjusted to values ranging from 3.0 to 9.0 ± 0.2 using either 0.10 M NaOH or 0.1 M HCl followed by shaking for 24 h. using an automatic shaker with speed 150 rpm. Finally, the final pH of the solution was measured and then plotted against the initial pH and the intersection point of the obtained curve was chosen to be the point of zero charge, pH_PZC_ of the RPNS sample.

### 3.6. Plackett-Burman Design (PBD)

In the current study, AWs and MWCNTs were investigated as adsorbents for the removal of the veterinary FQ antibiotic (SARA) from the contaminated water samples. PBD was the design of choice for optimizing the removal efficiency of the tested adsorbents. Four independent variables (pH, [SARA], AD, and CT) were varied as per the variable limits stated in Table 3. Two dependent variables; %R and *q_e_*, (mg/g) were measured as a function of the four variables. The design pattern implicated conducting 20 basic runs (comprising eight Ct Pt) in one replicate over two blocks. The factorial limits which were chosen carefully in order to get the maximum responses as well as the full design matrix are shown in Table 3 and Table 4 [28,29,42]. Experimental values for dependent variables were calculated using the formulas described by Equations (1) and (2), and the obtained values are listed in Table 4. Predicted values as obtained by Minitab^®^19 are also listed in Table 4. A comparison between the experimental and theoretical values was performed based on the values of the relative error (RE).

## 4. Conclusions

The present work has elaborated on the efficiency of waste-derived materials as potential adsorbents for the antibiotic SARA from contaminated water samples. A comparison between upcycled waste materials derived from pistachio nutshells (PNS) and *Aloe vera* leaves (AV) both raw and thermally treated from one hand and the MWCNTs on the other hand was conducted. Plackett-Burman design (PBD) was executed to decide upon variables’ levels and significance on two responses (%R and *q_e_*). Four variables were therefore considered; pH, [SARA] initial concentration, AD and CT. Statistical analysis proved that the adsorption of SARA was suppressed by increasing the pH and that the region of pH 5.0–6.0 is the region where maximum adsorption could be observed. Morphological and textural characterization of adsorbents’ surface showed that the surface of waste-derived materials in their raw format had a plenty of functional moieties, in contrast to the thermally treated adsorbents and the MWCNTs. This observation together with the BET analysis show that adsorption in case of raw waste materials would probably be attributed to chemisorption. However, physisorption cannot be excluded. In case of MWCNTs, adsorption was mainly physisorption. Equilibrium studies as indicated by the Temkin isotherm show that the adsorption of SARA onto RPNS goes over two stages; chemisorption to form one layer (confirmed by Langmuir isotherm) and then a physical interaction to form multilayers. Freundlich isotherm shows that the adsorption of SARA onto RPNS was irreversible and favorable. Adsorption kinetics was best-fitted to the pseudo-second-order model, and adsorption isotherms were best described by the Elovich model. All in all, both RPNS and MWCNTs were proved to be efficient adsorbents for SARA with MWCNTs being superior with a %R of 96.20%.

## Figures and Tables

**Table 1:** Reported adsorption – based methods for removal of SARA.

Adsorbent	Analytical Approach Used	Kinetics Model	Isotherm Model	Surface Area (m^2^/g)	*q_e_* (mg/g)	%R	References
Raw pistachio nutshells (RPNS) [A] Thermally treated PNS at 500 °C (TTPNS500) [B] Air-dried *Aloe vera* leaves (ADAV) [C] Thermally treated AV leaves at 500 °C (TTAV500) [D] Multi-walled carbon nanotubes (MWCNTs) [E]	Plackett-Burman design (PBD)	PSO *	Freundlich	4.24 [A] 76.94 [B] 3.94 [C] 7.56 [D] 482 [E]	49.75 [A]	82.39% [A] 96.20% [E]	Current approach
Powdered activated carbon (PAC)	Univariate analysis	PSO *	Freundlich	ND **	120	80–96%	[20]
Molecularly imprinted polymer nanoparticles (nanoMCN@MIPs) [A] Nonimprinted polymer nanoparticles NanoMCN@NIPs [B] Mesoporous carbon nanoparticles MCNs [C]	Univariate analysis	ND **	ND **	126 [A] 102 [B] 622 [C]	ND **	Deionized water “>90% [A] 30-40% [B and C]” Spiked seawater “>90% [A]”	[22]
Polydopamine-coated graphene oxide/Fe_3_O_4_ (PDA@ GO/Fe_3_O_4_) imprinted nanoparticles coupled with magnetic separation	Univariate analysis	PSO *	Langmuir	50.34	70.9	>95%	[23]
Magnetized metal-organic framework (MOF) (Fe_3_O_4_/MIL-101(Fe))	Response surface methodology (RSM)	ND **	Langmuir	1624.91	81.31	>93%	[24]
MIL-101(Cr)–SO_3_H a stable mesoporous MOF with polar –SO_3_H groups in the structure	Univariate analysis	ND **	ND **	ND **	898.2	ND **	[25]

* PSO: Pseudo second order, ** ND: Not determined.

**Table 2:** An assessment of the performance of the four adsorbents obtained by upcycling of the AWs in terms of %R and *q_e_*. The experimental conditions are pH = 7.00 ± 0.20, initial drug concentration [SARA] = 40 ppm, adsorbent dose (AD) = 50 mg/15 mL, and contact time (CT) = 30 min. The obtained values of %R and *q_e_* shown were determined utilizing Equations (1) and (2).

Candidate Adsorbent	%R	*q_e_* (mg/g)
Upcycled AWs		
RPNS	63.45	7.614
TTPNS500	7.871	0.944
ADAV	29.15	3.498
TTAV500	2.101	0.252

**Table 3:** Assessed factors together with their upper (+1), lower (−1) and mid-levels.

Factors	−1	0	+1
pH (A, pH unit)	5.0	7.0	9.0
Adsorbent Dose (AD, B, mg/15 mL)	20.0	50.0	80.0
Initial Drug Concentration ([SARA], C, ppm)	10.0	25.0	40.0
Contact Time (CT, D, min.)	10.0	65.5	120.0

**Table 4:** Experimental (measured) and predicted values of the two responses using RPNS and MWCNTs as adsorbents. Difference between experimental and predicted values is expressed as the relative error (RE).

Variables					RPNS						MWCNTs					
Trial No	pH	AD	[SARA]	CT	%R Obs. *	%R Pred. **	RE ***	*q_e_* Obs. *	*q_e_* Pred. **	RE ***	%R Obs. *	%R Pred. **	RE ***	*q_e_* Obs. *	*q_e_* Pred. **	RE ***
01	9(+)	20(-)	10(-)	10(-)	26.70	24.89	0.07	1.15	1.09	0.05	38.73	39.66	0.02	2.05	2.05	0
02	9(+)	20(-)	40(+)	10(-)	21.75	23.66	0.08	4.22	4.02	0.05	33.55	32.48	0.03	6.47	6.53	0.01
03	5(-)	20(-)	10(-)	120(+)	58.04	56.67	0.02	3.60	3.47	0.04	96.20	97.19	0.01	6.59	6.46	0.02
04	7(0)	50(0)	25(0)	65(0)	47.29	47.09	0.004	2.36	2.29	0.03	73.97	70.5	0.05	3.60	3.59	0.00
05	9(+)	80(+)	40(+)	10(-)	41.48	40.69	0.02	2.10	2.03	0.03	37.16	36.61	0.01	1.65	1.64	0.01
06	5(-)	80(+)	10(-)	10(-)	75.51	79.25	0.05	1.08	1.00	0.08	87.69	87.79	0.00	1.10	1.12	0.02
07	9(+)	80(+)	10(-)	120(+)	46.22	44.15	0.05	0.67	0.60	0.12	61.36	59.43	0.03	0.77	0.75	0.03
08	5(-)	80(+)	40(+)	10(-)	79.00	77.04	0.03	3.99	3.83	0.04	82.26	83.13	0.01	3.64	3.55	0.03
09	7(0)	50(0)	25(0)	65(0)	49.12	47.09	0.04	2.45	2.29	0.07	71.94	70.5	0.02	3.60	3.59	0.00
10	9(+)	80(+)	10(-)	120(+)	40.60	44.15	0.08	0.65	0.60	0.08	58.97	59.43	0.01	0.74	0.75	0.01
11	5(-)	20(-)	40(+)	120(+)	51.74	54.81	0.06	7.92	8.18	0.03	94.77	92.78	0.02	19.95	20.52	0.03
12	7(0)	50(0)	25(0)	65(0)	48.35	47.09	0.03	2.20	2.29	0.04	68.75	70.5	0.02	3.61	3.59	0.01
13	7(0)	50(0)	25(0)	65(0)	45.19	47.09	0.04	2.10	2.29	0.08	70.40	70.5	0.00	3.57	3.59	0.01
14	5(-)	20(-)	10(-)	10(-)	55.50	54.57	0.02	2.33	2.45	0.05	85.75	85.18	0.01	4.49	4.45	0.01
15	7(0)	50(0)	25(0)	65(0)	46.84	47.09	0.01	2.19	2.29	0.04	68.34	70.5	0.03	3.51	3.59	0.02
16	7(0)	50(0)	25(0)	65(0)	49.72	47.09	0.05	2.21	2.29	0.03	70.76	70.5	0.00	3.54	3.59	0.01
17	7(0)	50(0)	25(0)	65(0)	45.78	47.09	0.03	2.18	2.29	0.05	72.13	70.5	0.02	3.61	3.59	0.06
18	7(0)	50(0)	25(0)	65(0)	44.59	47.09	0.05	2.19	2.29	0.04	67.59	70.5	0.04	3.68	3.59	0.03
19	5(-)	80(+)	40(+)	120(+)	82.39	79.53	0.03	4.93	5.11	0.03	94.67	95.27	0.01	5.14	5.16	0.00
20	9(+)	20(-)	40(+)	120(+)	25.85	25.05	0.03	5.17	5.32	0.03	48.21	50.34	0.04	9.64	9.50	0.01

* Obs: observed readings; ** Pred.: predicted readings; *** RE = ǀ (Measured value - Actual value)/Actual value ǀ.

**Table 5:** Analysis of variance (ANOVA) for the transformed responses for both adsorbents.

RPNS										
Response	%R					*q_e_*				
Source	DF *	Adj SS *	Adj MS *	F-Value	*P*-Value	DF *	Adj SS *	Adj MS *	F-Value	*P*-Value
Model	2	24.1383	12.0692	345.40	0.000	6	3.65834	0.60972	5342.19	0.000
Blocks						1	0.01266	0.01266	110.94	0.000
Linear	2	24.1383	12.0692	345.40	0.000	4	2.77354	0.69339	6075.22	0.000
pH	1	17.2545	17.2545	493.80	0.000	1	0.58908	0.58908	5161.32	0.000
AD	1	6.8838	6.8838	197.01	0.000	1	0.69691	0.69691	6106.10	0.000
[SARA]						1	1.95224	1.95224	17104.89	0.000
CT						1	0.18325	0.18325	1605.59	0.000
Error	17	0.0398	0.0398	1.15	0.300	13	0.04748	0.04748	416.02	0.000
Curvature	1	0.5940	0.0349			1	0.00148	0.00011		
Lack-of-Fit	8	0.3342	0.0418	1.52	0.284	6	0.00037	0.00006	0.38	0.868
Pure Error	8	0.2200	0.0275			7	0.00112	0.00016		
Total	19	24.7323				19	3.65982			
**MWCNTs**										
**Response**	**%R**					***q_e_***				
Source	DF *	Adj SS *	Adj MS *	F-Value	*P*-Value	DF *	Adj SS *	Adj MS *	F-Value	*P*-Value
Model	5	968610	193722	347.94	0.000	5	11.9604	2.39209	7091.84	0.000
Linear	4	968590	242147	434.91	0.000	4	11.9160	2.97899	8831.84	0.000
pH	1	863285	863285	1550.52	0.000	1	1.7798	1.77984	5276.69	0.000
AD	1	3976	3976	7.14	0.018	1	5.7161	5.71612	16946.62	0.000
[SARA]	1	12530	12530	22.51	0.000	1	4.0004	4.00044	11860.15	0.000
CT	1	88799	88799	159.49	0.000	1	0.4196	0.41957	1243.89	0.000
Curvature	1	20	20	0.04	0.852	1	0.0445	0.04448	131.86	0.000
Error	14	7795	557			14	0.0047	0.00034		
Lack-of-Fit	6	2173	362	0.52	0.783	6	0.0026	0.00043	1.60	0.262
Pure Error	8	5622	703			8	0.0021	0.00027		
Total	19	976405				19	11.9652			

*DF is degrees of freedom, SS is sum of squares, and MS is mean of squares.

**Table 6:** Raman and Brunauer-Emmett-Teller (BET) analyses of RPNS, TTPNS500, ADAV, TTAV500 and thermally treated RPAL.

Parameters	RPNS	TTPNS500	ADAV	TTAV500	MWCNTs
**Raman I_D_: I_G_ Ratio**	0.61	0.65	0.24	0.67	0.11
**Langmuir surface area (SA) (m^2^/g)**	4.24	76.94	3.94	7.56	482.01
**Total pore volume (cm^3^/g)**	0.0082	0.0072	0.0112	0.0261	1.0778
**Average pore radius (°A)**	61.7	89.8	98.6	80.8	52.8

**Table 7:** General and linearized equation of Langmuir, Freundlich, Temkin and Dubinin-Radushkevich isotherms, beside their parameters for the adsorption of SARA on RPNS.

Isotherm	Equations (Generalized/ Linearized Forms)	Parameters	Value	
**Langmuir**	$q_{e}=\frac{q_{m}\;K_{L}\;C_{e}}{1-K_{L}\;C_{e}}$ $\frac{C_{e}}{q_{e}}=\frac{1}{q_{m}\;K_{L}}+\frac{C_{e}}{q_{m}}$	$q_{m}$ (mg/g)	49.75	
		$K_{L}$ (L·mole^−1^)	0.0314	
		R^2^	0.975	
**Freundlich**	$q_{e}=K_{F}C_{e}^{\frac{1}{n}}$ $\log\left(q_{e}\right)=\log\left(K_{F}\right)+\left(\frac{1}{n}\right)\log\left(C_{e}\right)$	1n	0.651	
		$K_{F}$ (mole/g) (L/mole)^1/n^	2.509	
		R^2^	0.9785	
**Temkin**	$q_{e}=\frac{RT}{b_{T}}\;\ln\left(A_{T}\;C_{e}\right)$ $q_{e}=\frac{RT}{b_{T}}\ln\left(A_{T}\right)+\frac{RT}{b_{T}}\ln\left(C_{e}\right)$	$b_{T}$ (J/mole)	250.0	45.54
		$A_{T}$ (L/mole)	0.389	0.025
		R^2^	0.964	0.984
**DR**	$\ln(q_{e})=\ln\left(q_{m}\right)-\beta \epsilon^{2}$	β	1.7 × 10^−8^	1.98 × 10^−9^
	$\epsilon =RT\left(1+\frac{1}{C_{e}}\right)$	E (kJ/mole)	5.423	15.891
		$q_{m}$ (mg/g)	48.04	6.23
	E=12β	R^2^	0.984	

**Table 8:** The kinetics study results corresponding to Figure 12.

Models	Parameter	Value	
Pseudo-first order (PFO) $\ln\left(q_{e}-q_{t}\right)=\ln\left(q_{e}\right)-k_{1}t$	K_1_ (min^−1^)	0.021	
	*q_e_* (mg/g)	8.868	
	R^2^	0.889	
Pseudo-second order (PSO) $\frac{t}{qe}=\;\frac{1}{k_{2}q_{e}^{2}}+\frac{1}{q_{e}}t$ *Where K_2_ is rate constant (g·mg^−1^·min^−1^)*	K_2_ *(*g·mg^−1^·min^−1^*)*	0.162	
	*q_e_*(mg/g)	13.94	
	R^2^	0.999	
Elovich equation is $q_{t}=\frac{1}{\beta }\ln\left(\alpha \beta \right)+\frac{1}{\beta }\ln\left(t\right)$ is used to predict the sorption mechanism, where q_t_ is adsorbed quantity at time t; while α and β are initial sorption concentration rate (mg·g^−1^·min^−1^), and desorption constant (g/mg), respectively.	Α	7.1 × 10^3^	
	Β	0.392	
	R^2^	0.978	
Weber-Morris intraparticle diffusion model is used to study the formed layers around the adsorbent and rate-controlling step, which is expressed as $q_{t}=K_{I}t^{0.5}+C$, where K_I_ is intraparticle diffusion rate constant (mg·g^−1^·min^−0.5^), and C is the boundary thickness effect.	K_I_	2.19	0.55
	C	19.02	26.16
	R^2^	0.934	0.898

## Abbreviations

Activated carbons (ACs); Adsorption capacity (*q_e_*); Adsorbent dose (AD); Air-dried *Aloe vera* leaves (ADAV); *Aloe vera* leaves (AV); Analysis of variance (ANOVA); Anderson-Darling statistic (A-D); Agro-wastes (AWs); Brunauer–Emmett–Teller (BET); Carbon nanotubes (CNTs); Central points (Ct Pt); Coefficient of determination (R^2^); Concentration of sarafloxacin ([SARA]); Confidence interval (CI); Contact time (CT); Degrees of freedom (DF); Deoxyribonucleic acid (DNA); Dubinin-Radushkevich (DR); Fluoroquinolone antibiotics (FQs); Fourier transfer infrared spectroscopy (FT-IR spectroscopy); Initial drug concentration ([SARA]) (C); International Union of Pure and Applied Chemistry (IUPAC); Langmuir Surface Area (SA); Magnetized metal-organic framework (MOF) (Fe_3_O_4_/MIL-101(Fe)); Mean of Squares (MS); Mesoporous carbon nanoparticles (MCNs); Molecularly imprinted polymer nanoparticles (NanoMCN@MIPs); Multi-walled carbon nanotubes (MWCNTs); Nonimprinted polymer nanoparticles (NanoMCN@NIPs); Percentage removal (%R); Pistachio nutshells (PNS); Plackett-Burman design (PBD); Point-of-zero-charge (PZC); Polydopamine-coated graphene oxide/Fe_3_O_4_ (PDA@GO/Fe_3_O_4_); Powdered activated carbon (PAC); Pseudo-first order (PFO); Pseudo-second order (PSO); Raw pistachio nutshells (RPNS); Relative error (RE); Sarafloxacin (SARA); Scanning electron microscope (SEM); Sum of squares (SS); Thermally treated AV leaves at 500 °C (TTAV500); Thermally treated PNS at 500 °C (TTPNS500); Thermogravimetric analysis (TGA); Wastewater treatment plants (WWTPs); Weber-Morris (WM).
